# Heavy Metals and Microplastics as Emerging Contaminants in Bangladesh’s River Systems: Evidence from Urban–Industrial Corridors

**DOI:** 10.3390/toxics13090803

**Published:** 2025-09-22

**Authors:** Raju Kumar Das, Mongsathowai Marma, Al Mizan, Gang Chen, Md Shahin Alam

**Affiliations:** 1 Department of Textile Engineering, University of Scholars, Dhaka 1213, Bangladesh; 2 Department of Mechanical and Production Engineering, Ahsanullah University of Science and Technology, Dhaka 1208, Bangladesh; 3 Department of Leather Engineering, Khulna University of Engineering & Technology, Khulna 9203, Bangladesh; 4 Department of Civil and Environmental Engineering, Florida State University, Tallahassee, FL 32310, USA

**Keywords:** bioaccumulation, Buriganga, Dhaleshwari, emerging contaminants, heavy metals, microplastics

## Abstract

Urban industrialization is a major driver of water pollution, particularly through emerging contaminants that pose significant health risks for humans and ecosystems. This critical review focuses on Bangladesh’s Buriganga and Dhaleshwari rivers, which pass through highly industrialized and urban areas, analyzing contaminant types, sources, pathways, and impacts. By synthesizing data from studies published between 2005 and 2024, the paper examines pollutants such as heavy metals (e.g., Cr, Cd, Pb, Ni, Zn, Hg, As, Mn, Cu, Fe) and microplastics in water, sediments, and biota. The Buriganga River shows extreme heavy metal contamination, with surface water Cr concentrations reaching up to 167,160 μg/L, Pb up to 3830 μg/L, and Fe up to 30,000 μg/L, and sediment Cr up to 4249 μg/g, Pb up to 3312 μg/g, and Fe up to 15,435 μg/g. In contrast, the Dhaleshwari River exhibits elevated but comparatively lower heavy metal concentrations in surface water (e.g., Cr up to 3350 μg/L; Cd up to 1890 μg/L; Pb up to 1320 μg/L; Ni up to 1732 μg/L; Fe up to 6040 μg/L) and sediments (Cr up to 282 μg/g; Fe up to 14,375 μg/g). Microplastic contamination in Buriganga is widespread across water, sediments, and biota and dominated by polymers such as polyethylene (PE), polypropylene (PP), and polyethylene terephthalate (PET). Industrial discharges, particularly from the textile, leather, and metal processing industries, are identified as primary sources for heavy metals and microplastics. Additional inputs from domestic waste, agricultural runoff, and municipal sewage intensify pollution, with Cr, Cd, and Pb notably frequently exceeding safety thresholds. Microplastics, originating from municipal waste and atmospheric deposition, persist in these rivers, posing ecological and public health risks. The persistence and bioaccumulation of heavy metals and microplastics threaten aquatic biodiversity by disrupting food chains and pose significant risks to local communities that depend on these rivers for agriculture, fishing, and daily water use. This review highlights the urgent need for comprehensive bioaccumulation studies, long-term monitoring, and enhanced detection techniques to better assess contamination levels. Strengthening environmental regulations, improving waste management, and adopting sustainable industrial practices are critical to mitigating emerging contaminant impacts and safeguarding these vital river ecosystems and public health.

## 1. Introduction

Rivers have long been demonstrated as crucial ecosystems because they supply essential amenities, including irrigation for agriculture, water for domestic and industrial use, and a habitat for aquatic organisms. However, the rapid industrialization and urbanization near water bodies in the last century have turned many rivers into pollution hubs, leading to extensive ecological deterioration. This transformation has been primarily driven by the unregulated release of pollutants from industrial and densely populated urban areas. Moreover, urban rivers have been impacted by the discharge of large volumes of untreated or inadequately treated industrial effluents containing complex contaminants [1]. Emerging contaminants (ECs), including heavy metals (HMs) like chromium (Cr), lead (Pb), nickel (Ni), iron (Fe), cadmium (Cd), copper (Cu), zinc (Zn), etc.; microplastics (MPs); pharmaceuticals and personal care products (PPCPs); pesticides; per- and polyfluoroalkyl substances (PFAS); endocrine-disrupting chemicals (EDCs); and flame retardants, have recently gained increasing attention in both the scientific and legislative arenas [2,3]. The critical difference between ECs and traditional contaminants is that the ECs are frequently found at low concentrations (usually between ng/L and µg/L) [4]. However, their enduring existence, capacity to bioaccumulate, and capacity to have long-lasting, subtle effects on ecosystems significantly impact human and environmental health [5,6]. Managing the diverse array of ECs is a growing challenge due to the lack of standardized procedures and regulatory frameworks for their identification and measurement. In developing countries like Bangladesh, weak policies covering industrial wastewater worsen the risks of EC persistence and bioaccumulation [7]. Many ECs persist through conventional water/wastewater treatment processes, prevailing in riverine systems [8,9]. Given the hydrological connectivity of aquatic ecosystems, the environmental footprint of ECs extends beyond local regions, raising both local and global concerns. Industrial pollution, particularly in rapidly developing nations, exemplifies the complex ecological challenges arising from the intersection of urban growth, industrial expansion, and inadequate regulation [10]. In Bangladesh, the Buriganga and Dhaleshwari rivers, which flow through the heart of Dhaka, are severely affected by untreated industrial effluents and other sources of ECs. These rivers are surrounded by major industrial hubs, including the areas of Narayanganj, Keraniganj, and Savar, which together house nearly two-thirds of the country’s industrial facilities [11]. Key industries in this region, such as tanneries, textile and dyeing factories, and metal processing units, are among the dominant contributors of ECs to the rivers [10,12,13]. Although these industries are now legally required to install effluent treatment plants (ETPs) and assess treatment efficiency for ECs, for decades before enforcement, untreated effluents were directly discharged into the Buriganga and Dhaleshwari rivers [11], leaving a legacy of contamination.

Complex chemical mixers in industrial effluents are the primary sources of pollution in the Buriganga and Dhaleshwari rivers, alongside municipal waste and untreated sewage [14]. Among these, the leather tanning industry (tanneries) is a primary and dominant polluter, historically responsible for over 60% of the waste entering the Buriganga and Dhaleshwari rivers [15]. In addition, recent research indicates a concerning level of adverse PFAS compounds in Dhaka’s surface and drinking water, mostly attributed to industrial effluents from the leather and textile industries [16]. Until 2017, the Hazaribagh tannery industries directly discharged their effluents and solid waste into the Buriganga without treatment [17]. Though the tanneries were later relocated to Savar, the shift only transferred the pollution burden to the Dhaleshwari River, especially since the common effluent treatment plant (CETP) remained nonfunctional until 2020 [13,18,19]. Moreover, textile industries release large amounts of dye, toxic chemicals such as azo dyes, HMs like chromium (Cr), and MPs at elevated concentrations. In contrast, the metal processing industry contributes to HM contamination involving Cr, lead (Pb), cadmium (Cd), nickel (Ni), mercury (Hg), arsenic (As), Cupper (Cu), iron (Fe), and zinc (Zn), which are difficult to remove by conventional wastewater treatment methods [20,21]. Similarly, electronic waste processing has intensified pollution involving Pb, Cd, polycyclic aromatic hydrocarbons (PAHs), persistent organic pollutants (POPs), and different types of MPs [22,23]. The cumulative effect of these pollutants has resulted in the Buriganga River becoming one of the most polluted rivers in Bangladesh, with significant ecological degradation and the loss of traditional functions like fishing and irrigation. The Dhaleshwari River, which supports local agriculture and fisheries, has also been adversely impacted. Contaminated irrigation water affects crop quality and poses risks to food safety. High concentrations of HMs, MPs, dyes, pigments, solvents, and surfactants in aquatic environments degrade biodiversity and threaten human health. Elevated levels of toxic elements have been detected in fish and other species from both rivers, raising concerns over carcinogenicity, skin disorders, respiratory issues, and waterborne diseases among local communities through the food chain [24,25,26]. The respiratory problems associated with these pollutants may occur through both direct and indirect routes. The primary pathway is indirect exposure via fish consumption, which may gradually damage respiratory organs. For instance, long-term exposure to Pb, Hg, and Cd has been linked to coughing, wheezing, shortness of breath, and impaired lung function [27,28,29].

Addressing the environmental health of the Buriganga and Dhaleshwari rivers is critical to safeguarding the well-being and livelihoods of the local population, necessitating a comprehensive approach that combines environmental protection with socioeconomic development. With a particular focus on the Buriganga and Dhaleshwari rivers, this critical review addresses the issue of ECs in industrialized urban rivers, with precise emphasis on HMs and MPs, their sources, and impacts on human and ecosystem health. Given Bangladesh’s scarcity of comprehensive EC studies, this review aims to consolidate existing knowledge, highlight research, and propose future directions. Ultimately, it contributes to the broader discourse on sustainable industrial practices, water quality management, and environmental health in rapidly urbanizing and industrializing nations.

## 2. Methodology

### 2.1. Geographic and Socioeconomic Context

**Figure 1:**
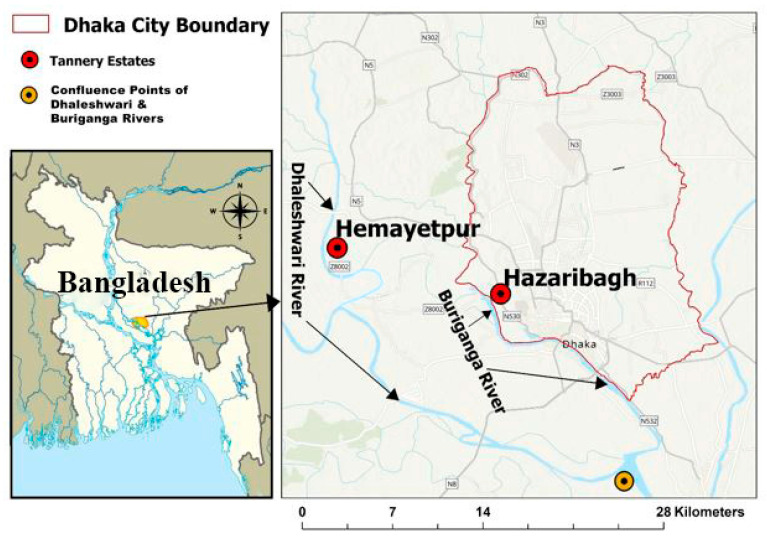
Geographical location of the Buriganga and Dhaleshwari rivers in Dhaka, Bangladesh.

Dhaka, Bangladesh’s capital, which has a population of over 18 million people, has seen rapid industrial and urban growth, severely affecting its rivers [30,31]. The Buriganga and Dhaleshwari rivers are central to the region’s economy, culture, and ecology, supporting agriculture, fishing, and industrial activities. The Dhaleshwari, a tributary of the Jamuna River, flows for about 160 km through the districts of Manikganj, Tangail, and Dhaka. In contrast, the 27 km Buriganga flows along Dhaka’s western boundary (Figure 1). Both lie in flood-prone zones, where seasonal inundation impacts water supply and agriculture. Low-income communities relying on these rivers are especially vulnerable to environmental stresses.

### 2.2. Systematic Critical Review

**Figure 2:**
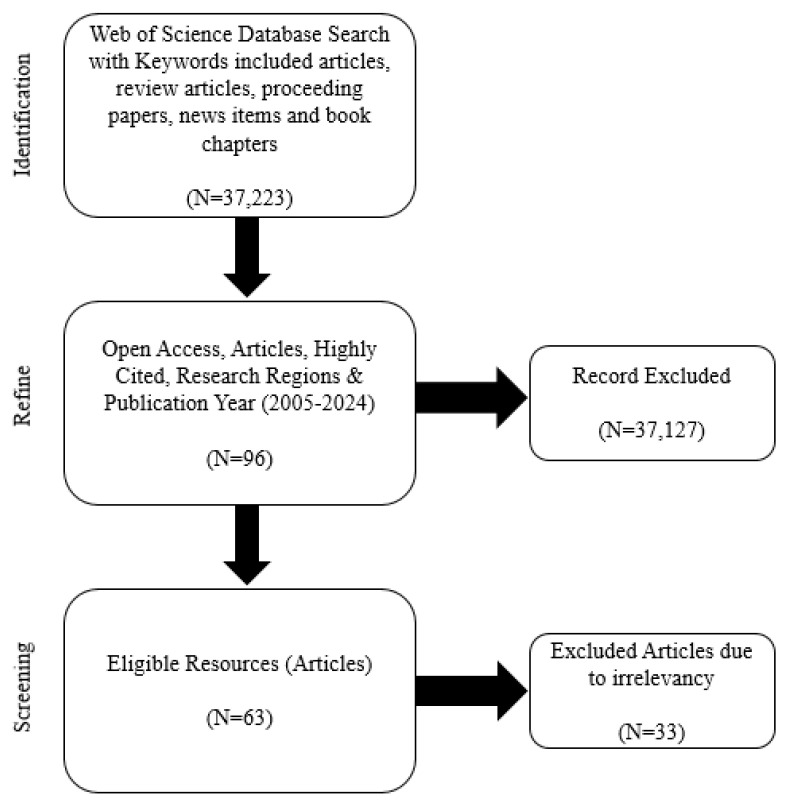
Flowchart of literature selection and screening process from the Web of Science database.

This study comprises a bibliometric and critical review of contaminants in Bangladesh’s Buriganga and Dhaleshwari rivers using the Web of Science (WoS) database (searched in July 2024). Keywords included “Buriganga River Pollution”, “Dhaleshwari River Pollution”, “Microplastics”, “PFAS”, “Heavy metals”, and “Bioaccumulation”, connected with the “OR” function. An initial 37,223 records were found, including research articles, review articles, conference proceedings, news items, and book chapters. After refining the search by publication type, language (English), country, citation metrics, and date range (2005–2024), 96 relevant articles were identified. Following abstract and title screening, 33 were excluded for irrelevance, resulting in a review of 63 articles focusing on microplastics, heavy metals, organic pollutants, and bioaccumulation in the Buriganga and Dhaleshwari rivers (Figure 2).

### 2.3. Bibliometric Analysis

**Figure 3:**
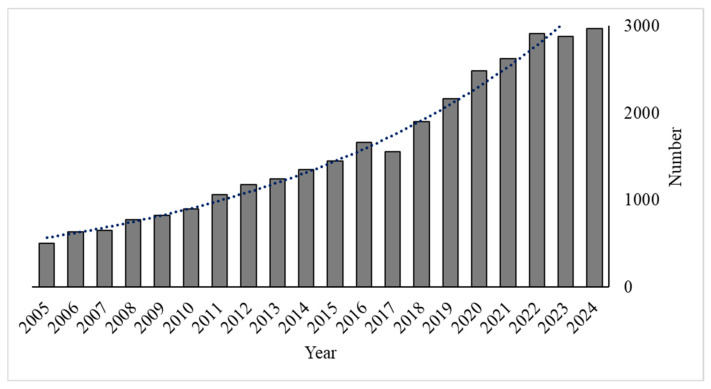
Trends in emerging contaminants on surface water: relevant Web of Science publications (2005–2024).

Bibliometrics has recently become increasingly popular as an effective method to estimate patterns in the evolution of research [2,32]. This research used VOSviewer (v1.6.20) to analyze citation metrics, publication outputs, and collaboration patterns from 2005 to 2024. Annual publication counts were assessed to identify growth trends, while citation analysis highlighted influential articles, authors, and journals in ECs research. Keyword co-occurrence mapping revealed thematic developments. Since 2011, over 1000 articles on ECs in surface water have been published annually, indicating a consistent upward trend (Figure 3).

**Figure 4:**
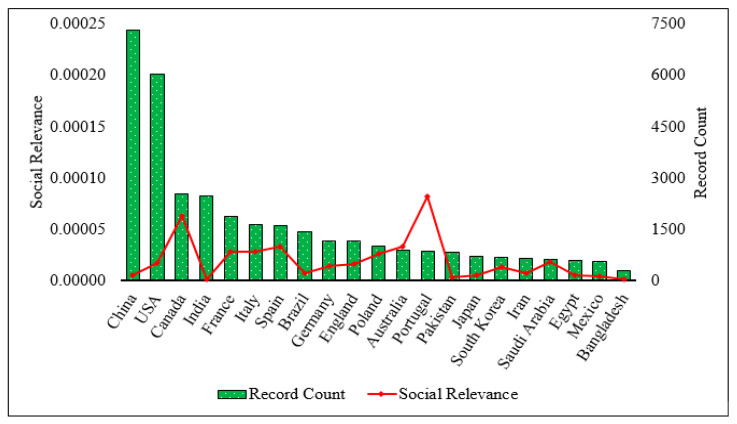
Statistics on published articles and social relevance by countries related to ECs (2005–2024) in Web of Science.

This comprehensive study highlights the many noteworthy scientific advances regarding ECs in surface water over the past two decades. Here, the social relevance (the ratio of the number of published articles and the total population in a specific country) is shown in Figure 4 to correlate the research strength of a nation in the EC in surface water field. China led with 7321 publications (with a low social relevance (SR) value), followed by the United States, which secured 6035 articles and an SR value of 0.00002, which was comparatively higher than China. Canada published 2531 articles with the second-highest SR value. This research area has become the global focus, with contributions from different countries like Brazil, Italy, India, Spain, France, Pakistan, Saudi Arabia, Germany, Egypt, Poland, England, South Korea, Iran, Australia, Portugal, Mexico, and Japan. Each country published over 500 articles, indicating significant involvement in this field; however, Portugal shows the highest SR value (0.00008) among the mentioned countries/nations. Bangladesh only produced 292 articles in the last two decades and had the lowest SR value, reflecting limited but growing engagement in the field (Figure 4).

## 3. Results

### 3.1. Bibliometric Analysis

**Figure 5:**
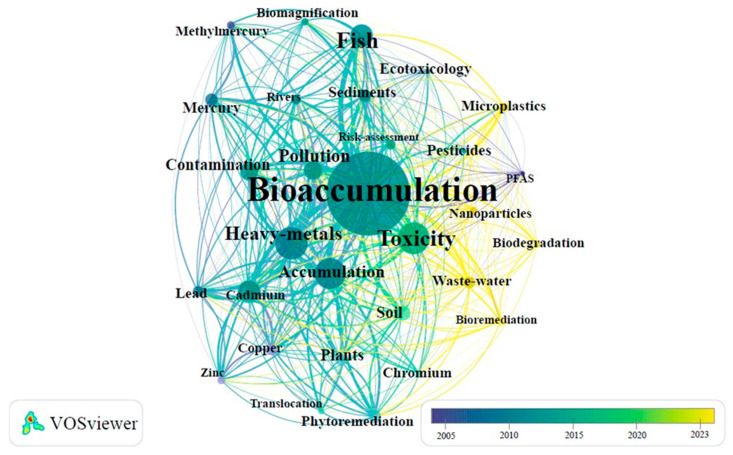
The co-occurrence analysis of the 30 most used keywords in ECs on surface water-related publications using association strength (2005–2024).

The use of specific keywords in related studies reflects prevailing research interests. A bibliometric analysis of the top 30 authors’ keywords revealed frequent terms such as “bioaccumulation”, “heavy metals”, “toxicity”, “fish”, “cadmium”, “phytoremediation”, “risk assessment”, “microplastics”, “pollution”, “biomagnification”, “bioremediation”, “lead”, “sediment”, “soil”, “contamination”, “translocation”, “copper”, “mercury”, “rivers”, “nanoparticles”, “chromium”, “biodegradation”, “zinc”, “wastewater”, “plants”, “ecotoxicology”, “accumulation”, “pesticides”, “methylmercury”, and “PFAS”. These keywords highlight a broad focus on contaminants in surface water research, particularly in areas such as bioremediation, wastewater management, biodegradation, and emerging pollutants like nanoparticles, PFAS, and microplastics (Figure 5). The thematic evaluation suggests ongoing research attention to topics such as “bioaccumulation”, “heavy metals”, “pollution”, “contamination”, “toxicity”, “wastewater”, “microplastics”, “fish”, “biodegradation”, “bioremediation”, and “nanoparticles”.

### 3.2. Occurrence of Heavy Metals (HMs)

HMs enter the environment (e.g., water, soil, and air) from anthropogenic and natural sources [13]. The Buriganga and Dhaleshwari rivers are affected by urbanization, and discharges from untreated or partially treated industrial and municipal sources exacerbate HM pollution [11]. Numerous studies have examined the HM levels in the surface water [14,33,34,35], river sediments [36,37,38,39], plants [24,40,41], and aquatic species, mainly fish [42,43,44,45], in the Buriganga and Dhaleshwari rivers, focusing on metals such as Cr, Pb, Cd, Ni, Zn, Hg, As, Mn, Cu, and Fe (Table 1). Studies reveal that the Buriganga and Dhaleshwari rivers are heavily contaminated with multiple pollutants, as the surface-water concentrations of heavy metals often surpass national and international standards (e.g., Buriganga Cr 35,330–167,160 μg/L; Dhaleshwari Cr 2590–3350 μg/L, though some later studies reported safer ranges of 0.1–3.17 μg/L) [24,46,47]. Sediments, plants, and fish also show marked bioaccumulation of Cr, Pb, Cd, Ni, and Zn, frequently exceeding Average Share Value (ASV), Toxicity Reference Value (TRV), Lowest Effect Level (LEL), and Severe Effect Level (SEL) and WHO/FAO thresholds [26,29,37,38,41,43,45]. To assess bioaccumulation in fish, tissues such as muscle, liver, the gills, stomach, and intestine have been analyzed, providing a comprehensive overview of metal distribution and accumulation (Table 4). However, the continuous influx of industrial, agricultural, and domestic wastes introduces diverse organic and inorganic pollutants, with rivers acting as major conduits for pollutant transport and ecosystem degradation [41].

**Table 1:** Comparison of heavy metal concentrations in Buriganga and Dhaleshwari river surface water (μg/L), number of samples, and sample collection period (wet weight). The concentration ranges reflect the lowest and highest values reported in the references.

Sampling Period	Number of Samples	Cr	Cd	Pb	Ni	Zn	Hg	As	Mn	Cu	Fe	References
**Buriganga River**												
Sum 2010	264	35,330–167,160	10–70	1180–3830	-	-	40–360	-	120–1920	-	-	[48]
Sum and Win 2017	-	19–57	ND—5	ND—114	38–80	50–670	5–421	ND	-	160–565	-	[12]
November 2014–December 2015	-	9.8–13	<3.0	15–21	0.55–10.85	8.36–14.77	-	<3–8.25	-	<5.0	-	[46]
-	-	2–270	-	1	-	1000–4000	<1	-	-	-	-	[49]
August 2019–February 2020	210	27.7	12.7	29.9	22.2	65.1	-	16.5	238	26	-	[50]
March 2010	30	12–180	30–90	100–210	90–400	110–900	-	2–220	60–310	100–990	90–2200	[51]
Sum and Win in 2018–2019	20	1990	-	280	1050	1060	-	-	-	690	1231	[26]
-	16	-	-	58	-	113	-	10	230	94	1400	[52]
-	40	59	22.9	182	136	23	-	192	-	-	-	
March–May 2014	-	-	100–500	300–500	2500–3500	60,000–90,000	-	-	150,000–200,000	1500–2500	20,000–30,000	[33]
Mon and Win 2021–2022	168	128–170	48.7–79.1	65–97.2	112–151	201–273	20.1–27.7	46.6–77.6	134–167	132–186	478–620	[53]
Sum and Win in 2009	20	1430–1960	160–220	230–500	150–170	220–260	-	240–400	-	1710–2740	-	[54]
April 2016	10	365	3.19	40.66	-	660	1.61	-	-	-	1230	[55]
Pre-Mon, Mon and Post-Mon	-	BDL-224	BDL-1	BDL-24	8–27	20–120	13–270	-	-	-	-	[41]
Over 2011	84	2.2–20	-	30–254	-	-	-	-	-	-	-	[35]
December 2017 and January 2018	78	0.57–3.17	0.004–0.07	0.004–0.08	1.22–3.18	3.14–19.92	BDL–0.02	2.70–5.27	0.11–96.59	-	1.91–12.78	[56]
December 2017 and January 2018	72	0.1–3.17	BDL—0.07	0.004–0.16	0.22–3.18	2.27–19.92	BDL—0.02	0.72–5.83	BDL–96.59	0.38–9.05	0.68–12.78	[57]
**Dhaleshwari River**												
September 2022	8	177	24	122	BDL	-	-	-	-	-	-	[29]
Mon, Post-Mon, and Win in 2018–2019	32	20–900	2	50	20	10–30	-	-	-	10	490–6040	[47]
Mon, post-Mon, and Win in 2018–2019	24	40–370	2	5	20	5–20	-	-	-	10	2440–2730	[58]
April 2021–January 2022	30	1704–2215	276–422	343–456	1057–1732	344–438	14–16	164–184	3336–4509	614–1506	190–341	[31,59]
-	-	710	190	-	620	180	-	-	-	-	-	[14]
Mon and Win	-	2590–3350	1520–1890	1020–1320	-	-	-	410–750	-	1010–1090	-	[24]
-	41	-	130–420	630–3900	-	1600–5490	-	-	-	-	-	[34]
October 2018	-	653	-	225	395	1341	-	-	-	-	-	[45]
August–September 2019	-	0.18–0.64	0.27–0.45	-	-	6.68–8.17	-	2.81–3.01	-	-	-	[57]
**Standard Value (μg/L)**												
WHO		50	3	10	20	3000	1	10	500	2000	300	[60]
Bangladesh (Drinking water)		50	3	10	50	5000	1	50	400	1500	300–1000	[61]
Bangladesh (Industrial Effluents)		500	2000	100	1000	5000	10	200	2000	3000	3000	[61]

BDL—Below Detection Limit, ND—Not Detected, Sum—Summer (March–June), Win—Winter (December–February), Mon—Monsoon (July–September).

#### 3.2.1. Chromium (Cr)

Chromium is a common element in the Earth’s crust and is primarily found in trivalent (Cr^3+^) and hexavalent (Cr^6+^) forms in the environment [62]. Across the Buriganga and Dhaleshwari rivers, Cr concentrations in surface water, sediment, plants, and fish show significant variability and often exceed permissible limits set by the World Health Organization (WHO) and Bangladeshi standards for drinking water and industrial effluents (Table 1). In Buriganga, the Cr levels in surface water have ranged from 35,330 to 167,160 μg/L in 2010 [48] to as low as 0.1–3.17 μg/L in the period 2017–2018 [57]. The elevated levels in 2010 are attributed to the untreated discharge of tannery effluents from Hazaribagh, while the reduction after 2016 is likely linked to the relocation of the tannery industry to Savar. Seasonal studies have reported an average Cr concentration of 1990 μg/L, which is still above the WHO (50 μg/L) and Bangladesh (500 μg/L) effluent standards [26]. Other studies have found concentrations ranging from 57 to 1960 μg/L, while some reported levels within safe limits, between 0.1 and 27.7 μg/L (Table 1). In the Dhaleshwari River, the highest Cr levels in surface water were 2590–3350 μg/L during the monsoon and winter seasons [24], whereas the lowest Cr concentration of 0.18–0.64 μg/L was reported [57] via the use of a new multi-branch Integrated Catchment (INCA) model.

In the Buriganga River’s sediments, the Cr concentration found by Mohiuddin and Ogawa [54] ranged from 105 to 4249 μg/g in the summer and winter seasons of 2009, while the lowest was found [12], ranging from 21.22 to 22.47 μg/g. In Dhaleshwari River, the highest Cr concentration of 186 μg/g was observed [38]. Only a few studies are available on the Dhaleshwari River; however, they consistently reported Cr levels above guideline thresholds such as ASV, TRV, LEL, and SEL (Table 2).

**Table 2:** Comparison of Buriganga and Dhaleshwari river heavy metals in sediments (μg/g), number of samples, and sample collection period (dry weight). The concentration ranges reflect the lowest and highest values reported in the references.

Sampling Period	Number of Samples	Cr	Cd	Pb	Ni	Zn	Hg	As	Mn	Cu	Fe	References
**Buriganga River**												
Sum and Win, 2017	-	21.22–22.47	0.27–0.28	107.76–110.64	19.71–21.04	208.777–222.30	0.61–0.62	2.03–2.15	-	159.07–168.96	-	[12]
-	5	101.2	0.82	79.4	-	502.26	-	-	-	184.52	-	[63]
Mar 2010	15	1019–1884	4.21–11.28	50.12–80.2	35–59	45.45–60.5	-	11.82–26.4	368–692	50.12–80.2	9480–15,435	[51]
Sum and Win, 2018–2019	20	106	-	17	33	29	-	-	-	31	4655	[26]
February–March 2013 and August–September 2013	36	17–841	2–19	43–3312	62–539	-	-	7.6–67	-	62–712	-	[37]
Sum and Win, 2009	20	105–4249	3.5–9.5	56–1592	56–244	129–3002	-	9–34	-	53–743	-	[54]
Pre-Mon, Mon, Post-Mon	-	BDL—103.58	BDL < −0.16	2–8.9	BDL–21.93	26.2–71.9	-	-	-	-	-	[41]
7 August 2015 and 5 February 2016	7	39.70–41.45	0.21–0.23	10.41–11.40	6.39–7.14	36.73–40.71	0.001–0.02	0.18–0.21	-	14.07–15.93	37.58–39.06	[64]
-	9	119–2050	-	<0.36–93	-	61.0–210	0.09	0.91–23	423–701	<1.95–83.3	2.21–2.82	[39]
**Dhaleshwari River**												
Sum	5	BDL—282.4	BDL—4.4	BDL—414.6	85.1–264.5	-	-	-	-	-	11,800–14,375	[36]
Mon and Win	-	96.02–118.37	1.56–1.67	23.69–26	-	-	-	5.15–6.59	-	21.15–24.15	-	[24]
September 2019	24	186	-	8.78	-	-	-	-	3.12	1.76	42.7	[38]
**Standard Value** **(μg/g)**												
ASV ^a^		90	0.3	20	68	95	0.4	13	850	45	47,200	[65]
TRV ^b^		26	0.6	31	16	110	-	6	-	16	-	[66]
LEL ^c^		26	0.6	31	16	120	0.2	6	460	16	2%	[67]
SEL ^d^		110	10	250	75	820	2	33	1100	110	4%	[67]

ASV ^a^ (Average Share Value), TRV ^b^ (Toxicity Reference Value), LEL ^c^ (Lowest Effect Level), and SEL ^d^ (Severe Effect Level), BDL—Below Detection Limit, Sum—Summer (March–June), Win—Winter (December–February), Mon—Monsoon (July–September).

Cr accumulation in plants along the Buriganga River ranged from 5 to 32 μg/g, exceeding the WHO/FAO’s permissible limit of 2.3 μg/g [40]. The highest Cr content of 211.70–270.55 μg/g [29], while the lowest range of 1.06–10.7 μg/g in 27 plant samples from the Dhaleshwari River [13] (Table 3). Fish samples from both rivers showed Cr concentrations ranging from 0.34 to 187.07 μg/g, significantly surpassing the WHO guideline of 0.05 μg/g (Table 4). The highest Cr levels in fish (87.01–187.07 μg/g) were recorded in the Buriganga River during January–February 2018 and May–June 2019 [26].

#### 3.2.2. Cadmium (Cd)

Cadmium (Cd) is a non-essential transition metal with high toxicity that poses potential health risks for plants, humans, and animals. It bioaccumulates in plants and transfers into the human body through food chains and has a 25–30 y half-life [68]. According to the Drinking Water Standard for Bangladesh and WHO, the concentration of Cd should not surpass 3 μg/L (Table 1). However, the Cd concentration in surface water in the Buriganga and Dhaleshwari rivers exceeded the allowable limit [24,33,59]. For example, Rampley et al. (2020) [56] collected 78 water samples from different locations in the Buriganga River and found Cd concentration ranged from 0.004 to 0.07 μg/L, well below the WHO and Bangladeshi standards. Similarly, another study by Whitehead et al. (2019) [57] on the Dhaleshwari River reported slightly higher Cd concentrations of 0.27–0.45 μg/L, which were also lower than acceptable limits. In contrast [33] observed much higher Cd concentrations in the Buriganga River surface waters, ranging from 100 to 500 μg/L (Table 1), and Lipy et al. (2021) [24] found extremely high concentrations of 1520 to 1890 μg/L in the Dhaleshwari River, far exceeding the permissible thresholds.

Sediment analyses from both rivers revealed a slight variation between the observed Cd concentrations and standard allowable limits. For the Buriganga River sediment, Cd concentrations ranged from 4.21 to 11.28 μg/g, with the lowest value being below the detection limit (BDL) of 0.16 μg/g [41,51]. In the Dhaleshwari River, Cd concentrations in sediments ranged from 1.56 to 1.67 μg/g, surpassing the ASV (0.3 μg/g), TRV (0.6 μg/g), and LEL (0.6 μg/g) thresholds, but not exceeding the SEL value (10 μg/g) [24] (Table 2). The Cd levels in plant samples studied from the Buriganga and Dhaleshwari rivers across seasons (i.e., pre-monsoon, monsoon, and post-monsoon) and years were concerning. In Buriganga, the concentrations ranged from 0.01 to 0.32 μg/g, while in the Dhaleshwari they were between 0.58 and 3.42 μg/g, exceeding the WHO/FAO permissible limit of 0.05 μg/g (Table 3). Similar trends were observed in fish samples (Table 4), where the Cd concentrations exceeded the WHO standard of 0.05 μg/g, raising significant public health concerns. For instance, Baki et al. (2020) [46] reported Cd concentrations ranging from less than 0.1 to 13.43 μg/g in fish samples collected from different locations in the Buriganga River, highlighting the elevated risk of Cd exposure through the food chain.

#### 3.2.3. Lead (Pb)

Pb has been widely acknowledged as a hazardous environmental contaminant and a significant threat to public health, particularly in developing countries where unregulated industrialization is common [69]. Across the Buriganga and Dhaleshwari Rivers, Pb concentrations in water, sediment, plants, and fish have often been reported to be above national and international standards (Table 1). In the Buriganga River, the highest Pb content was found to be 1180–5160 μg/L in the water samples [48], which was almost 100–500% higher than the WHO and Bangladeshi standards. The lowest Pb concentration was recorded at 0.004 to 0.08 μg/L [56]. In contrast, by analyzing 24 water samples collected from the Dhaleshwari River in the monsoon, post-monsoon, and winter seasons of 2018–2019, Hasan [58] found a Pb concentration of 5 μg/L, which is below the allowed limit (10 μg/L) set by WHO. According to the ASV, TRV, LEL, and SEL standards, the Pb concentration should not exceed 20, 31, 31, and 250 μg/g in river sediment samples, respectively (Table 2). Some studies found that Pb accumulation exceeded the permissible limits, while very few showed that Pb values were below the allowable limit. For instance, Islam [37] investigated 36 sediment samples from the Buriganga River in 2013, finding Pb concentrations varying between 43 and 3312 μg/g, surpassing the standard values (Table 2). On the other hand, Pb content was recorded to be highest (414.6 μg/g) in the sediments of the Dhaleshwari River in the dry season [36], highlighting severe Pb pollution in different seasons. A similar Pb accumulation trend in the 0.25 to 82.89 μg/g range was noticed in plants grown on the Buriganga and Dhaleshwari rivers, which exceeded the WHO/FAO recommended values of 0.1 μg/g (Table 3). In addition, significant differences were experienced for Pb concentrations in different fish samples at various locations in both rivers (Table 4). The situation is more alarming for Pb accumulation in the fish samples of the Buriganga and Dhaleshwari rivers due to the high concentration (0.27–18.79 μg/g) compared to the WHO maximum allowable limit of 0.2 μg/g (Table 4).

#### 3.2.4. Nickel (Ni)

Ni is a well-known HM found at very low environmental concentrations, especially in soil. It is released into the environment through various industrial activities, including nickel mining, nickel–cadmium battery industries, incinerations, power plant processes, rubber and plastic industry activities, and electroplating processes [70]. The widespread application of nickel across multiple industries leads to occupational exposure that seriously impacts human health and fosters further environmental pollution, disturbing the ecosystem [70]. According to the WHO standards, the maximum concentration of Ni should not surpass 20 μg/L in drinking water (Table 1). In contrast, Bangladesh permits limiting the presence of Ni in drinking water up to 50 μg/L and 1000 μg/L for industrial discharge (Table 1). Studies carried out in the Buriganga and Dhaleshwari rivers reported that the average Ni concentration in surface water ranges between 0.22–3500 μg/L and 0–1732 μg/L, respectively (Table 1). The maximum Ni concentration of 3500 μg/L was found [33] in the Buriganga River from water samples collected in March–May 2014. On the other hand, Whitehead [57] found Ni concentrations in surface water samples collected from the Buriganga River of 0.22–3.18 μg/L, below the permissible limit (Table 1). The level of Ni in sediments in both rivers was concerning, as the maximum and minimum ranged from 6.39 to 539 μg/g and 85 to 265 μg/g in the Buriganga and Dhaleshwari rivers, respectively (Table 2). This indicates that the maximum values for both the Buriganga and Dhaleshwari river sediments exceeded the ASV (68 μg/g), TRV (16 μg/g), LEL (16 μg/g), and SEL (75 μg/g) values. Furthermore, the level of Ni in plant samples from the Buriganga River varied from 0.05 to 27.75 μg/g, and for the Dhaleshwari River they varied from 0.20 to 22.87 μg/g; the allowable limit set by the WHO/FAO is 10 μg/g (Table 3). Baki et al. (2020) observed the highest range of Ni content (8.66–90.49 μg/g) in collected fish samples from the Buriganga River, while Dhaleshwari River fish exhibited a concentration of 0.5–8.5 μg/g, exceeding the WHO-recommended value (0.5 μg/g) [45,46].

#### 3.2.5. Zinc (Zn)

Zn is beneficial in the human body and other biological entities. Although Zn has historically been considered relatively non-toxic, recent research indicates that free ionic zinc (Zn^2+^) can be highly toxic to neurons, glial cells, and other cell types when it exceeds safe levels [71]. Zn toxicity in aquatic environments is influenced by abiotic factors such as pH, dissolved organic matter, and hardness [72]. WHO and Bangladesh recommend the highest permissible Zn concentrations for drinking water to be 3000 and 5000 μg/L, respectively. Kibria [33] observed the highest Zn concentration, which ranged between 60,000 and 90,000 μg/L in the Buriganga River water samples, which was 18–30 times higher than the WHO standard. However, many studies found that the status of Zn contamination in the Buriganga and Dhaleshwari rivers was under control, as the values were under the recommended limits (Table 1). The lowest Zn content (6.68–8.17 μg/L) for water samples was reported in the Dhaleshwari River [57].

Zn levels in sediment samples were observed at 26.2 to 3002 μg/g for the Buriganga River, which was quite alarming (Table 2). In contrast, no studies have investigated the Zn content in the Dhaleshwari River (Table 2). In the summer and winter seasons of 2018–2019, Hossain [26] observed the lowest Zn concentration of 29 μg/g from 20 sediment samples of the Buriganga River at different depths, which was below the ASV (95 μg/g), TRV (110 μg/g), LEL (120 μg/g), and SEL (820 μg/g) values. On the other hand, only a few studies were found that investigated the Zn levels in plants in the adjacent territories of both rivers; however, the concentration of Zn varied between 6 and 244 μg/g (Buriganga River) and 58 and 118 μg/g (Dhaleshwari River), exceeding the WHO/FAO standard value of 9.4 μg/g (Table 3). Additionally, the Zn concentration in river fish is also considered significant, indicating the toxicity level of river pollution. Notably, the maximum Zn concentration (314.47 μg/g) was found by studying fish samples from the Dhaleshwari River [45]; 271.33 μg/g was the highest Zn concentration reported in the Buriganga River, surpassing the allowable limit set by WHO (50 μg/g) [73]. However, the lowest range of Zn concentrations in fish samples found in the Buriganga River was 11.08 to 35.12 μg/g (Table 4) [26].

#### 3.2.6. Mercury (Hg)

Mercury (Hg) is a dangerous environmental pollutant recognized as a significant concern by various global metal monitoring organizations. This metal exists in aquatic systems in elemental, inorganic, and organic forms [74]. Toxicology studies also prove that Hg, especially methyl mercury (MeHg), is very toxic to the human embryo and fetus [75]. MeHg is produced mainly through microbial methylation in aquatic systems. It bioaccumulates and biomagnifies in food chains, posing severe risks to ecosystems and human health [76]. The limit for Hg in drinking water has been set at 1 μg/L by the WHO and Bangladeshi standards. The highest Hg concentration in Buriganga and Dhaleshwari river water varied between 40 and 590 μg/L and 14 and 16 μg/L, respectively (Table 1), surpassing WHO and Bangladeshi standards. On the other hand, Rampley [56] observed the lowest Hg concentration (in the range of 0–0.02 μg/L) in collected water samples among all other studies, which was below the permissible limit (1 μg/L). Sediment samples in the Buriganga River ranged from 0.001 to 0.09 μg/g, levels much lower than the standard ASV (0.4 μg/g), LEL (0.2 μg/g), and SEL (2 μg/g) values (Table 2), indicating Hg pollution in the Buriganga River sediment was under control. Unlike the Buriganga River, no mercury concentration was measured or recorded for sediment samples from the Dhaleshwari River (Table 2). Some traceable amounts of Hg were seen in the fish of the Buriganga and Dhaleshwari rivers, ranging from 0.33 to 1.36 μg/g, where the permissible limit is 0.5 μg/g (WHO), indicating potential risks to humans for the consumption of fish from these rivers (Table 4).

#### 3.2.7. Arsenic (As)

Arsenic (As) is a harmful metalloid that enters the environment through natural and human activities. While trace amounts can be found in living organisms, exposure to high concentrations poses serious health risks and has toxic effects on the human body [77]. The permissible limit of As set by WHO is 10 μg/L, which is much lower than Bangladeshi standards for drinking water (50 μg/L). Inorganic arsenic (iAs), mainly originating from mineral deposits, mining, and industrial effluents, contaminates groundwater, rivers, and crops. Human exposure occurs primarily through drinking contaminated water and consuming food grown in polluted areas, leading to cancer, cardiovascular diseases, skin lesions, and neurological effects [77]. The status of As in the Buriganga and Dhaleshwari rivers is concerning, as many studies have observed that the As concentration in both rivers surpasses the WHO and Bangladeshi standards, making the river water unable to be used for different purposes (Table 1). Based on the concentration reported by many studies, the concentration of As in water samples was determined as 0–400 μg/L for the Buriganga River and 2.81–750 μg/L for the Dhaleshwari River, respectively (Table 1). In addition, Whitehead [57] showed the lowest As content (0.72–5.83 μg/L) based on 72 water samples analyzed from different locations of the Buriganga River, which was below the recommended limit suggested by WHO. For the Dhaleshwari River, the study found the lowest concentration of As to be between 2.81 and 3.01 μg/L in 2017, 2018, and 2019.

**Table 3:** Comparison of plants’ heavy metals (μg/g), number of samples, and sample collection period (dry weight). The concentration ranges reflect the lowest and highest values reported in the references.

Sampling Period	Number of Samples	Cr	Cd	Pb	Ni	Zn	Hg	As	Mn	Cu	Fe	Reference
**Buriganga River**												
-	6	5–32	0.01–0.08	0.25–0.9	0.05–0.8	6–17	-	-	-	1–6.3	-	[40]
Pre-Mon, Mon, Post-Mon	-	0.95–163.1	0.23–0.32	1.29–6	0.66–27.75	18.68–244	-	-	-	-	-	[41]
**Dhaleshwari River**												
September 2022	13	211.70–270.55	1.24–3.42	6.21–82.89	9.75–22.87	-	-	-	-	-	-	[29]
-	27	1.06–10.7	0.58–1.21	0.45–1.65	0.20–0.35	58.3–118	-	-	-	2.79–4.59	-	[13]
**Standard Value (μg/g)**												
WHO/FAO	-	2.3	0.05	0.1	10	9.4	-	0.002	500	40	425.5	[78]

WHO—World Health Organization; FAO—Food and Agriculture Organization; Sum—Summer (March–June), Mon—Monsoon (July–September).

The concentration of HMs in sediment serves as a key indicator of overall HM contamination within the aquatic ecosystem [24]. Numerous studies have been carried out to assess the As concentration in the sediment samples from the Buriganga and Dhaleshwari rivers. The highest As concentrations in sediment samples from both rivers were 67 μg/g (Buriganga River), exceeding the standard values of ASV (13 μg/g), TRV (6 μg/g), LEL (6 μg/g), and SEL (36 μg/g). The highest As concentration for the Dhaleshwari River was 6.59 μg/g, which is below the standard values except for TRV (6 μg/g) and LEL (6 μg/g) (Table 2). Remarkably, no data on As accumulation in the plants surrounding the Buriganga and Dhaleshwari rivers was found (Table 3). Additionally, As contamination could be a severe threat to the fish in the Buriganga River. Many studies have found levels of As (0–3.99 μg/g) in river fish samples exceeding the permissible limit (0.001 μg/g) set by WHO (Table 4). Therefore, the consumption of fish from the Buriganga River poses a severe health concern. Only one study did not find any trace of As in the Dhaleshwari River fish samples [24].

#### 3.2.8. Manganese (Mn)

Mn is an indispensable element for various physiological processes in the human body, while an excessive amount of Mn in the human body, via drinking water or other pathways, causes symptoms of neurotoxicity [79]. The WHO recommends a maximum Mn concentration of 400 μg/L for drinking water, while the Environment Conservation Rules (ECRs) for Bangladesh set a limit of 500 μg/L. Kibria [33] found the highest level of Mn (150,000–200,000 μg/L) in the Buriganga River water samples analyzed from March to May 2014 (Table 1). However, a few studies found Mn concentrations in the Buriganga River under the permissible limit (Table 1) [50,52,53,56,57]. One study analyzed Mn concentrations in 30 water samples from the Dhaleshwari River and to be at concentration of 3336–4509 μg/L, significantly exceeding WHO and Bangladeshi standards [59]. Studies invoving sediment have shown Mn concentrations in both rivers ranging from 3.12 to 701 μg/g (Table 2), which are below the ASV (850 μg/g) and SEL (1100 μg/g) sediment quality guidelines but beyond the LEL values (450 μg/g), indicating a potential risk to sensitive aquatic organisms. However, compared to other HMs such as Pb and Hg, Mn contamination in these rivers poses relatively lower environmental risks. Similar to Hg and As, no research has been conducted to determine the Mn level in plants near either river (Table 3). While the Mn levels in the sediments of the Buriganga River are within permissible limits, the elevated concentration of Mn in fish samples from these rivers is a serious concern (Table 4). The WHO sets the maximum safe limit for Mn in fish at 0.01 μg/g. Still, studies have reported concentrations in fish from the Buriganga River exceeding this threshold value, sometimes finding values from 0.31 to 16.46 μg/g (Table 4). In contrast, no specific research is available that determines the Mn concentration in fish samples from the Dhaleshwari River (Table 4).

#### 3.2.9. Copper (Cu)

Cu is an essential element necessary for life and generating high-energy metabolites by acting as an important component of many proteins and enzymes [80]. Bangladesh’s drinking water and industrial effluent standards for Cu prescribe maximum acceptable concentrations of 1500 and 3000 μg/L, respectively. The Cu concentration in Buriganga River water samples from different sampling points and in different seasons and years varied between 5.0 and 2740 μg/L (Table 1). Furthermore, the Cu concentration in the Dhaleshwari River ranged from 10 to 1506 μg/L and did not exceed the WHO recommended value (maximum 2000 μg/L). The total amount of Cu in the sediments of the Buriganga and Dhaleshwari rivers ranged from 1.95 to 743 μg/g and 1.76 to 24.15 μg/g, respectively. Saha and Hossain [63] determined a Cu concentration of 184.52 μg/g in sediment samples, which surpassed the ASV, TRV, LEL, and SEL standard values. Similarly, studies conducted on Dhaleshwari River sediment samples did not exceed the ASV (45 μg/g) and SEL (110 μg/g) recommended values but surpassed the TRV (16 μg/g) and LEL (16 μg/g) standards (Table 2). In addition, for plant samples from the Buriganga and Dhaleshwari rivers, one study reported Cu in the range of 1.0–4.59 μg/g, which was within the acceptable limits set by WHO/FAO (40 μg/g), especially those used for food or fodder (Table 3). Studies on fish from the Buriganga and Dhaleshwari rivers showed that the Cu concentrations in fish tissues varied but were often within the acceptable limit of 30 μg/g recommended by WHO/FAO. The observed Cu concentration in fish samples ranged from 0.09 to47.3 μg/g in the Buriganga River and from 0.42 to 2.68 μg/g in the Dhaleshwari River (Table 4).

**Table 4:** Comparison of heavy metals in Buriganga and Dhaleshwari River fish (μg/g wet weight), number of samples, and sample collection period. The concentration ranges reflect the lowest and highest values reported in the references.

Sampling Period	Sampling Species	Analyzed Tissues	Number of Samples	Cr	Cd	Pb	Ni	Zn	Hg	As	Mn	Cu	Fe	Reference
**Buriganga**														
1 year in 2010	*Anabas testudineus*, *Channa punctata*, and *Oreochromis mossambicus*	Whole body	-	2.7	0.12	0.72	-	-	0.56	-	0.53	-	-	[48]
September 2015–December 2015	*Puntius ticto*, *Heteropneustes fossilis*, and	Liver, intestine, kidney, and muscles	-	0.51–7.75	<0.1–13.43	1.65–5.35	8.66–90.49	-	-	<0.1–0.12	-	0.09–31.04	-	[46]
	*Channa punctatus*													
April–	*Heteropneustes fossilis*	Muscle, gill, stomach,	30	1.40–8.65	0.3–5.50	1.79–18.79	-	16.82–67.8	-	0.2–3.99	-	6.22–47.3	-	[42]
May-11		intestine, and liver												
January–February 2018 and May–June 2019	*Heteropneustes fossilis*, *Channa punctatus*, and *Channa striata*	Muscle	12	87.01–187.07	-	1.2–5.07	0.07–3.01	11.08–35.12	-	-	-	2.07–3.51	19.66–39.07	[26]
November 2017	*Heteropnuestes*	Whole body	-	BDL–0.35	0.21–0.73	1.14–10.14	0.35–1.14	77.26–271.33	-	BDL	2.02–16.46	0.19–1.7	-	[73]
	*fossilis*, *Channa punctatus*, *Notopterus notopterus*, *Channa striata*, and *Colisa fasciata*													
November–December 2017	*Batasio batasio*, *Colisa fasciata*, *Gonialosa manmina*, *Amblypharyngodon microlepis*, *Awaous*	Edible portions	-	0.41–0.94	-	0.32–0.65	-	82.07–189.64	0.33–1.36	0.41–0.51	0.31–1.31	16.09–26.70	59.63–98.58	[44]
	*guamensis*, *Heteropneustes fossilis*, *Otolithoides*													
	*pama*, *Mastacembelus armatus*, *Labeo calbasu*, and *Channa punctata*													
Mon, Win, and Sum	*Labeo rohita*	Muscle	-	6.69–8.24	1.02–2.44	5.25–7.38	-	-	-	-	-	20.74–25.22	-	[81]
**Dhaleshwari**														
Mon and Win	*Labeo rohita*, *Catla*	Muscle tissue, guts, and gills	-	0.78–2.23	0.24–0.51	0.27–1.87	-	-	-	ND	-	0.42–2.68	-	[24]
	*catla,* and *Bagarius bagarius*													
October 2018	*Heteropneustes fossillis*, *Channa punctata*, *Nandus nandus, Chanda*	body (skin)	84	1.60–116.7	-	1.5–11.5	0.45–8.5	57.09–314.47	-	-	-	-	-	[45]
	*nama, Anabas testudineus, Mystus gulio,* and *Colisa fasciata*	scales, and muscle) and head (no gills)												
**Standard Value**														
WHO			-	0.05	0.05	0.2	0.5	50	0.5	0.001	0.01	30	50	[82]

BDL—Below Detection Limit; WHO—World Health Organization; Mon—Monsoon (July–September); Win—Winter (December–February); Sum—Summer (March–June); ND—Not Detectable.

#### 3.2.10. Iron (Fe)

Fe is an essential component of various metalloproteins and plays a critical role in key biochemical processes, including oxygen transport and sensing, catalysis, and electron transfer [83]. When present in excessive amounts, Fe can damage cells and tissues, making it essential to tightly regulate Fe levels in the body to maintain a proper balance (iron homeostasis) [84]. The WHO and ECRs set the permissible limit for Fe in drinking water at 300 and 300–1000 μg/L, respectively. Kibria [33] noticed the maximum Fe ranging from 20,000 to 30,000 μg/L in the analyzed water samples from the Buriganga River. In another study, the maximum Fe concentration of 6040 μg/L was observed based on 32 water samples of the Dhaleshwari River. Both of these values exceed the allowable limits (Table 1) [47]. Moreover, Whitehead [57] found the lowest range of Fe of 0.68–12.78 μg/L in Buriganga water, and the minimum Fe concentration of 190 μg/L of the Dhaleshwari river water [59](Table 1). The Fe content in the Dhaleshwari river sediment was found to be the highest (11,800–14,375 μg/g) in the dry season [36], which was under the ASV (47,200 μg/g) limit (Table 2). At the same time, Bhuiyan [51] observed an Fe concentration of 9480–15,435 μg/g based on 15 sediment samples from the Buriganga River, which was within the ASV value (47,200 μg/g). Despite the environmental concerns surrounding industrial pollution in the Buriganga and Dhaleshwari rivers, similar to Hg, As, and Mn, no studies have been conducted to precisely assess the Fe concentration in plants growing along the banks of these rivers (Table 3). Research indicated that the Fe concentrations in fish ranged from 19.66 to 39.07 μg/g [26], suggesting that this range is within acceptable limits recommended by WHO for human consumption. However, Jolly [44] reported the highest range of 59.63–98.58 μg/g, which exceeded the WHO allowable limit. No research has determined the Fe content in fish from the Dhaleshwari River (Table 4).

### 3.3. Occurrence of Microplastics (MPs)

MPs are emerging environmental pollutants in freshwater systems, marine habitats, the atmosphere, and terrestrial ecosystems [85]. MPs in water and sediment can later transfer into aquatic species such as fish, snails, and crabs. MPs can harm humans, inducing oxidative stress, metabolic disorders, immune responses, and neurotoxicity [86]. MPs can also release hazardous pollutants such as polybrominated diphenyl ethers (PBDEs), bisphenol A (BPA), phthalates, and nonylphenol, which have been strongly linked to cancer and various reproductive disorders in both humans and other organisms [87]. As a result, these micro-pollutants have become the most concerning contaminants worldwide. A study of the Buriganga River detected MPs at levels of 4.33–43.67 items/L in surface water and 17.33–133.67 items/kg in sediment samples (Table 5) [85]. On the other hand, MP contents in the ranges of 0.25–0.12 MPs/mL, 3.50–8.16 MPs/g, and 0.94–5.34 MPs/g for sediment, water, and aquatic species in the summer and winter seasons, respectively [87] (Table 5). Moreover, a study on Buriganga River sediment samples detected the concentration of MPs to be 165.45 ± 127.87 items/kg [43]. However, due to differences in sampling locations, as well as other influencing factors such as sampling season, methodology, extraction procedures, and identification techniques, different values were observed by various studies (Table 5).

Along with studies on the concentration of MPs, the types of microplastic polymers present are also crucial for understanding their sources, chemical risks, environmental impact, and potential threats to ecosystems and human health. More than 30 types of MP polymers have been identified in different studies involving the Buriganga River [43]. For example, Islam and Islam [85] Identified polymers such as Polyvinylchloride (PVC), Polystyrene (PS), Polyester, Polyethylene terephthalate (PET), and polyamide. One study identified the highest concentrations of the different polymers present in the Buriganga River, indicating the significant sources of microplastic pollution [87]. However, no studies have investigated the specific MP polymers in the Dhaleshwari River (Table 5).

**Table 5:** Comparison of MP concentrations, number of samples, and sample collection period in surface water, sediments, and aquatic animals. The concentration ranges reflect the lowest and highest values reported in the references.

Sampling Period	Number of Samples	Conc of Microplastics (MPs)	Type of Polymers	References
**Buriganga River**				
March 2021	11 (Sediment)	165.45 ± 127.87 items/kg (sediment)	Polyethylene (PE), Polypropylene (PP), and Polyethylene terephthalate (PET)	[43]
-	-	4.33–43.67 items/L (Surface water); 17.33–133.67 items/kg (Sediments)	Polystyrene (PS), Polyvinylchloride (PVC), Polyethylene terephthalate (PET), Polyester, and Polyamide	[85]
Sum and Win	9 (Water), 31 (Sediment), and 79 (Aquatic Species)	0.25–0.12 MPs/mL (Water); 3.50–8.16 MPs/g (Sediments); 0.94–5.34 MPs/g (Aquatic Species)	Ethylene-vinyl acetate (EVA), Polyethylene terephthalate (PETE), Acrylonitrile butadiene styrene (ABS), High-density polyethylene (HDPE), Cellulose acetate (CA), and Nylon	[87]

## 4. Discussion

### 4.1. Sources of Emerging Contaminants in Buriganga and Dhaleshawari

**Figure 6:**
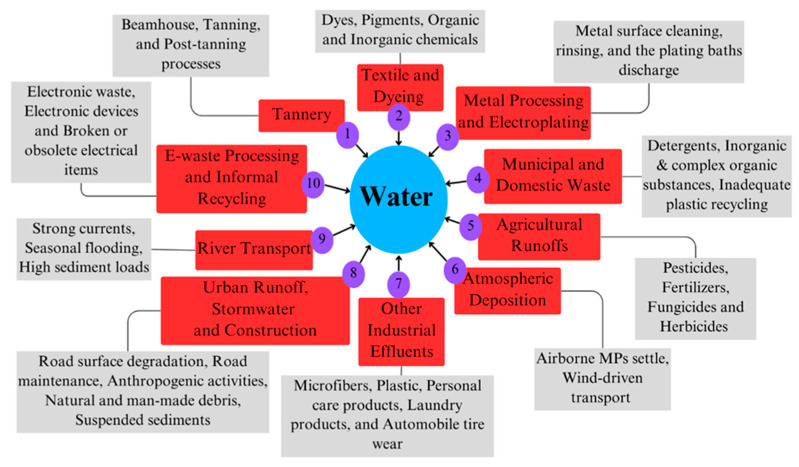
Sources of ECs in the Buriganga and Dhaleshwari rivers.

Inadequate waste management corresponding to the growing population and insufficient effluent treatment in Dhaka exacerbate the pollution of the Buriganga and Dhaleshwari rivers. The discharge of domestic and industrial solid and liquid waste, primarily into the Buriganga and Dhaleshwari rivers, intensifies the pollution load, as shown in Figure 6.

#### 4.1.1. Sources of Heavy Metals (HMs)

The primary sources of HM pollution in the Buriganga and Dhaleshwari rivers are tannery, textile, and dyeing factories, metal processing units, and municipal waste discharge. These activities release metals such as Cr, Pb, Cd, Ni, Hg, Zn, Mn, As, Cu, and Fe into the rivers [14,34,48].

##### Tannery Industries

Tanning involves processing hides and skins using a variety of chemicals, with basic chromium sulfate being the most prevalent [88]. Leather processing (beamhouse, tanning, and post-tanning) generates large amounts of Cr-containing waste and effluents, while related industries such as the manufacture of footwear, leather products, and paints also contribute toxic metals to nearby aquatic environments [18]. Tannery effluents contain significant chemical impurities, primarily HMs like Cr, Cd, Ni, Zn, Fe, and Cu. These substances are difficult to remove through conventional treatment methods, such as sedimentation, filtration, coagulation–flocculation, and aerobic/anaerobic biological treatment, and often remain in the discharged water, leading to surface and groundwater pollution [18,89]. For decades, most tanneries in Hazaribagh, Dhaka, have discharged untreated wastewater directly into the Buriganga River, severely degrading its water quality [48]. The Department of Environment (DoE) reported that around 334 tanneries in Hazaribagh and Rayer Bazar released nearly 22,000 m^3^ of toxic waste daily into the Buriganga River due to the absence of treatment facilities and weak enforcement. These operations, along with the Dhaka–Demra–Narayanganj industrial estate and the factories of Kamrangirchar in Dhaka, remained the primary sources of HM pollution for over 60 years [54]. In 2016, the tanneries were relocated to Hemayetpur, Savar, where a central effluent treatment plant (CETP) was established with a 25,000 m^3^/day capacity. However, frequent malfunctions released partially treated waste until 2020. Despite being fully operational now, past practices have already contributed significantly to the HM load in the water and sediments of the Buriganga and Dhaleshwari rivers, posing ongoing threats to biota [90].

##### Textile and Dyeing Industries

The textile and dyeing industries pose a significant threat to Bangladesh’s environmental and water quality, with the majority of these facilities situated along riverbanks to easily transport raw materials and finished goods. These operations use a wide array of dyes, pigments, and chemicals, generating substantial volumes of wastewater that are often discharged either untreated or only partially treated into the surrounding environment [91]. This polluted water spreads through nearby land, agricultural zones, and irrigation channels before ultimately entering surface water systems such as the Dhaleshwari River [10]. In 2016, Bangladesh’s textile industries manufactured 1.80 million metric tons of fabric, generating 217 million cubic meters of wastewater [92]. Wastewater from the textile processing industry typically exhibits high pH and TDS values, elevated concentrations of suspended solids (SS), and significant levels of chlorides and nitrates, as well as high levels of HMs, including Pb, Cd, Cu, Zn, Ni, Cr, Mn, Na, and Fe [10]. Some dyes (mordant, azo, metal complex, and synthetic dyes), fixing agents, and pigments, especially phthalocyanine-based pigments, are used to enhance color fastness, brightness, or durability in fabrics; these are the major source of HMs in the textiles and dyeing industries [20,93]. Moreover, effluents from handloom dyeing industries often consist of HMs, such as Fe, Zn, Cu, Cr, Cd, Mn, and Pb, exceeding both the inland surface water and irrigation water quality established by the Industrial Effluent Discharge Standards (IEDSs) [94].

##### Metal Processing and Electroplating Facilities

The electroplating and metal processing industries located along the riverbanks in Bangladesh produce high volumes of wastewater containing significant amounts of HMs, such as Cr, Ni, Cu, Zn, Pb, and Fe [95,96]. This contamination primarily results from metal surface cleaning and rinsing and the discharge of spent plating baths. The waste often exceeds the permissible limits. In addition, electroplating processes utilize metals such as Ni, Cd, Cu, Zn, Ag, Cr, and Pb. Approximately 4% of the total metal is lost as wastage, resulting in sludge and effluents. HMs primarily bind to suspended particles in the water and eventually settle into sediments, where they become immobilized; the rate of HM accumulation in sediments is influenced by various sediment properties, including texture, mineralogical composition, and pH, resulting in HM concentrations typically higher than those in water bodies [97]. Therefore, treating these effluents properly before discharging them into water bodies is essential to prevent further environmental degradation.

##### Municipal and Domestic Waste

Bangladesh’s urban rivers, like the Buriganga and the Dhaleshwari, are frequently used as industrial effluent and municipal sewage discharge points. Rapid, unplanned urbanization and poorly regulated industrial expansion in Dhaka have severely degraded the water quality of these rivers [14,98]. Moreover, the Buriganga River is a vital water source for densely populated catchments, supporting municipal, agricultural, domestic, and industrial needs [51]. In countries like Bangladesh and India, waste is often dumped openly without scientific management or prior treatment, contributing to significant environmental deterioration. Municipal solid waste typically contains biodegradable components (e.g., cellulose, lignin, starch, protein, and lipids) and various chemical pollutants such as detergents, inorganic substances, complex organics, and HMs [99]. Household municipal solid waste, for example, household dust, batteries, disposable household materials (e.g., metal parts, bottle tops, broken wire parts, etc.), plastics, inks, paints, body care products, medicines, and household pesticides, introduces HMs into the environment [100]. Open dumping sites, such as those in Savar near the Dhaleshwari River, lack proper infrastructure and financial support, exacerbating environmental damage. During monsoons and heavy rainfall, due to the biochemical activities of organic chemicals as a result of degradation, a liquid known as “leachate” is formed. Leachate is rich in organic content and also facilitates the solubilization and mobilization of HMs such as Cu, Pb, Zn, Mn, and Cd in the surrounding rivers [101]. Consequently, these metals might enter food crops, soil–water–air systems, and domestic animals, ultimately affecting human health [28].

##### Agricultural Runoff and Pesticide Use

Agricultural activities significantly contribute to HM contamination in river systems through runoff and pesticide application. The pesticides, fertilizers, and herbicides used in farming often contain HMs such as Pb, Cr, As, Co, and Ni, which can leach into nearby water bodies [102,103]. Significant levels of heavy metals in fertilizer are beneficial for increased yield. EU law, however, also specifies the maximum limit for several types of heavy metals in fertilizers bearing the CE mark, including Cd 3 mg/kg, Cr 150 mg/kg, Cu 230 mg/kg, Hg 1 mg/kg, Ni 90 mg/kg, Pb 150 mg/kg, and Zn 500 mg/kg [104]. In areas near the Dhaleshwari River, crop cultivation during the dry and summer seasons involves extensive pesticide application. These substances often reach rivers via surface runoff, contributing to contamination. Furthermore, phosphate fertilizers contain trace amounts of Cd and As that accumulate in soils and are transported to rivers through surface runoff and irrigation return flows [103]. According to recent studies, phosphatic fertilizers in Bangladesh may have nickel and cadmium levels that are around 2.5 and 4.2 times higher than the national allowable limits, respectively [105]. Pesticides and herbicides that enhance crop productivity may contain metal-based compounds that persist in the environment [28]. For instance, copper-based fungicides are often applied to prevent fungal infections, introducing significant amounts of copper into agricultural soils and eventually making their way into rivers. A comparative case from the White River in Muncie, Indiana (USA) illustrates that even in less industrialized watersheds, irrigation water quality fluctuates seasonally due to runoff and wastewater inputs, with up to 24.5% of samples unsuitable for irrigation during late summer [106]. In contrast, the Buriganga and Dhaleshwari are subjected to far higher loads of heavy metals from both agricultural and industrial sources, making the risks for irrigation, aquaculture, and aquatic ecosystem health even more critical. Importantly, when pollutant levels are evaluated against aquaculture water quality standards (e.g., FAO/WHO guidelines), the threats extend beyond irrigation and drinking water. Elevated concentrations of Cd, Pb, As, and Cu in these rivers exceed safe thresholds for fish farming, impairing fish growth, reproduction, and survival and leading to bioaccumulation in aquatic organisms. These contaminants ultimately compromise aquatic biodiversity and food safety, highlighting the dual challenge of maintaining agricultural productivity while safeguarding aquaculture and ecosystem health [24,36,73,107]. Implementing sustainable agricultural practices, such as controlled fertilizer application, buffer zones, and proper waste management, is crucial to mitigating HM pollution in the Buriganga and Dhaleshwari rivers.

##### Urban Runoff and Construction Activities

The rapid industrialization and unplanned urbanization of major cities in developing countries like Bangladesh contribute to HM pollution, which alters the properties of land, including affecting infiltration quality, decreasing soil permeability, and accelerating surface runoff [108]. The transportation of pollutants like SS and HMs, particularly Cr, Zn, Ni, Cu, and Pb, from urban environments to rivers via washing off during rainfall accounts for 75% (by dry weight) of pollutants originating directly or indirectly from traffic, atmospheric deposition, road surface degradation, road maintenance, and other anthropogenic activities [109,110]. Urban roads are a primary source of stormwater runoff and play a significant role in water quality and aquatic life degradation, mainly hampering the river ecosystem [109,110,111]. These toxic pollutants could also pose ecological risks, varying with pollutant loads, types, toxicity, and mobility. In addition to HMs, urban runoff contains significant amounts of hydrocarbons and human-origin bacteria, such as fecal coliforms, and alters pollution indices such as biochemical oxygen demand (BOD), dissolved oxygen (DO), chemical oxygen demand (COD), TSS, and total solids [110].

##### E-Waste Processing and Informal Recycling

E-waste refers to discarded electrical and electronic devices, including obsolete or damaged equipment such as computers, televisions, mobile phones, and circuit boards. These wastes contain a mix of hazardous elements, like beryllium (Be), As, Cd, Cr, Hg, Pb, chlorofluorocarbons, flame retardants, polybrominated diphenyl ethers, polycyclic aromatic hydrocarbons, and dioxin-like compounds, and non-hazardous elements, such as Cu, selenium (Se), and Zn, as well as precious metals like gold (Au), silver (Ag), and platinum (Pt) [112]. For instance, printed circuit boards (PCBs) typically contain about 16% Cu, 3% Fe, 2% Ni, and 0.05% Ag of total weight [113]. However, improper e-waste processing, like open burning, acid leaching, and manual dismantling, poses significant environmental risks [112]. Bangladesh has reportedly become one of Asia’s largest e-waste dumping sites, generating over 10 million metric tons of e-waste annually, both legally and illegally, due to the rapid expansion of digitalization [113]. A large portion is processed informally, particularly in makeshift workshops near the Buriganga and Dhaleshwari Rivers, where toxic residues from unregulated recycling enter water bodies and surrounding ecosystems. Studies in Lianjiang River (Guiyu) and Mandoli, India, both major informal e-waste processing areas, revealed substantial HM contamination (e.g., Pb, Cu, Cd, Ni, Zn, As, and Se) in water, soil, and plants due to these activities [112,114]. Similarly, the rivers in Bangladesh are increasingly affected by these operations, contributing significantly to the region’s HM pollution.

#### 4.1.2. Sources of Microplastics (MPs)

MPs are tiny plastic particles of 1 μm to 5 mm in size that originate from various sources and, once introduced into rivers, pose risks to aquatic life and public health [115]. MPs pose a more significant threat to the natural world than larger plastics [116]. Nearly 10% of all plastic litter in lakes and rivers is thought to eventually transform into MPs due to various external factors, including heat, UV radiation, and water [117]. It is critical to identify their origins to understand the formation and effects of MPs and develop mitigation methods [118]. Two types of MPs, primary and secondary, are essential to understanding MP sources [119]. Primary MPs are initially manufactured as small-sized plastics, such as plastic pellets, microfibers, and micro-beads. In contrast, secondary MPs are generated from the breakdown of larger plastic items through biogeochemical degradation processes.

##### Industrial Effluents

Industrial processes play a critical role in MP pollution, primarily through plastic manufacturing, personal care, chemical cleaning products, and wear and tear from automobile tires and material abrasion [120]. Industrial wastewater treatment plants (WWTPs) play a dual role; they act as partial barriers while simultaneously serving as notable sources of MPs [121]. Although advanced treatment technologies can achieve removal efficiencies of over 90%, residual MPs in treated effluents still represent a significant route for environmental contamination [120]. For instance, Xu and Hou [21] reported that untreated effluents from the textile dyeing industry contained an average of 334 MPs/L, which dropped to 16.3 MPs/L after treatment, achieving 95% removal efficiency. Long and Pan [122] observed influent concentrations ranging from 1.57 to 13.69 items/L, which declined to 0.20–1.73 items/L post-treatment, yielding efficiencies between 79.3% and 97.8%. Similarly, eleven major WWTPs in China exhibited influent MP concentrations of 196.0 ± 11.9 MPs/L and effluent levels of 9.0 ± 1.1 MPs/L [21], confirming removal rates exceeding 90%.

Despite such performances in some settings, significant challenges remain, especially in developing countries. For example, in Bangladesh, many WWTPs are poorly maintained or underperforming. A case study involving 11 textile industries reported microfiber removal efficiencies between 23.52% and 82.19% [123]. In another assessment of WWTPs from five sectors, i.e., washing, dyeing, printing, and pharmaceuticals, removal efficiencies ranged from 0 to 96%, 54 to 96%, 0 to 88%, and 45 to 100%, respectively [121], indicating inconsistent and insufficient removal capacity. Also, improper waste disposal, leakages, and direct discharge from industries significantly contribute to MP pollution in rivers such as the Buriganga and the Dhaleshwari. The daily inflow of industrial effluents has turned these rivers into “black water” zones, as reported by several researchers [85,124]. Furthermore, synthetic fabrics release microfibers during washing processes, which can bypass treatment systems and accumulate in aquatic environments [123]. The MPs detected in industrial wastewater vary in shape and composition, commonly including fibers, fragments, films, pellets, foams, and granules [121]. To effectively reduce MP pollution, technological advancements such as membrane filtration systems, which can remove up to 99.9% of MPs, offer a promising solution [125]. Implementing such technologies, especially in under-resourced settings, will be essential to address the growing threat of industrial microplastic contamination.

##### Municipal Waste and Poor Waste Management

Improper handling of municipal solid waste is a major contributor to MP contamination in surface water bodies, yet it remains an under-researched issue in Bangladesh [126]. The country, home to over 3000 plastic-related enterprises, has seen a sharp rise in plastic use, with per capita consumption growing from 2.07 kg in 2005 to 3.5 kg by 2014, resulting in around 3000 tons of plastic waste produced daily that accounts for 8% of the total solid waste [127]. The urban areas in Bangladesh produce 633,129 tons of plastic waste annually, with only 51% being recycled. This inadequate management ranks the country among the worst plastic polluters globally [127]. Bangladesh’s per capita plastic waste generation rate of 3.5 kg in 2024 is relatively low compared to many developed and developing countries [128]. However, it has the highest percentage (2.32%) of mismanaged plastic waste ending up in the ocean compared to other countries. This is attributed to inadequate recycling, a lack of awareness, weak governance, and the uncontrolled disposal of plastic waste in open areas, riversides, roadsides, or directly in water bodies [129]. Consequently, rivers such as the Buriganga and Dhaleshwari are heavily polluted with MPs. Similar patterns have been observed elsewhere, for instance, in Malaysia, where 20–30% of household waste is improperly disposed of near rivers, further emphasizing the global link between poor municipal waste handling and MP pollution [130]. If left unmanaged, even small communities can significantly contaminate freshwater resources through everyday consumer plastic waste [131]. Tackling this crisis requires a comprehensive approach that includes infrastructure development, adoption of new waste collection systems where the waste will be separated according to international standards, promotion of recycling technologies, strengthening the legal framework, establishing industrial processing zones, and raising public awareness in line with the efforts of neighboring developing and developed countries [128].

##### Agricultural Runoff

Modern agricultural practices contribute to MP contamination in soil through various methods, such as plastic mulching, soil amendments, sewage irrigation, littering, fertilizer coatings, and runoff. Agricultural communities in Bangladesh are especially vulnerable to MP exposure because of the lack of proper safety measures during farming practices [132]. Farmers use different types of polymers, such as low-density polyethylene (LDPE), polypropylene (PP), PS, PVC, etc., in different agricultural practices (e.g., mulching films, protecting plants, twine, fertilizers, and pesticide coating, Silage and bale nets, bottles, etc.) [133]. Organic manures are increasingly recognized as an emerging source of MP contamination in agricultural lands, affecting aquifer microorganisms and groundwater drinkers through the vertical transportation of MPs from farming soil [134]. This may result from agricultural runoff due to different factors, including natural disasters (e.g., floods, rains, landslides, etc.), which contribute to the introduction of plastics into water bodies. These plastics eventually degrade into MPs, following hydrological and sediment transport pathways influenced by surface morphology, topography, land use, etc. [124,134,135]. During heavy rainfall, flooding, and surface runoff, these MPs may be transported from agricultural lands into the Buriganga and Dhaleshwari rivers. Research on the Rhône River showed a surge in plastic transport shortly after rainfall, suggesting that surface runoff may play a more significant role in MPs accumulating in water bodies, mainly rivers, than other activities [136]. Terrain characteristics, land use, and surface morphology further influence the extent of this transport, emphasizing the need for sustainable agricultural practices and improved waste containment strategies to mitigate MP pollution in river basins.

##### Urban Runoff and Stormwater

Stormwater and urban runoff are considered major pathways for MP to enter aquatic ecosystems [137]. Urban stormwater runoff is a complex mixture of precipitation, natural and artificial debris, suspended sediments, and chemical pollutants that are washed off the urban landscape during rainfall. Among the different organic and inorganic contaminants, MPs have also been found in stormwater [109,110]. The Buriganga and Dhaleshwari rivers surround the Dhaka and Savar urban areas and are the largest water reservoirs. Therefore, all the MPs flow away to the rivers during rainfall or in stormwater or runoff, creating intense MP pollution in the aquatic ecosystem. A study claimed that urban runoff was one of the significant sources of MP pollution in the rivers surrounding Dhaka city, especially during the dry period (January–April) [43]. Additionally, the MP concentrations in inland waters varied significantly during rainfall and increased several-fold in the days following rain events [138]. This highlights that urban stormwater runoff is a key contributor to artificial debris, like MPs, in aquatic systems [139]. Studies have reported MP concentrations of 5.4–10 particles/L in urban stormwater across three urban catchments, emphasizing the role of stormwater as a key transport medium for MPs into receiving water bodies [139]. However, research on the contribution of MPs from street runoff and urban runoff to riverine and coastal pollution remains limited [137].

##### Atmospheric Deposition

Atmospheric deposition is an increasingly recognized pathway contributing to MP accumulation in aquatic systems such as the Buriganga and Dhaleshwari rivers. This process involves the deposition of airborne MPs onto land and water surfaces over time. The atmosphere is an effective transport route, allowing lightweight and fine plastic particles to remain suspended and travel significant distances before settling [25,140]. Due to their buoyant and minuscule nature, MPs can be widely dispersed by wind, reaching both densely populated zones and remote regions. Unlike localized inputs from surface runoff, atmospheric fallout distributes MPs over broader spatial scales, compounding the complexity of contamination [141,142]. In Bangladesh, the urban centers and industrial zones surrounding the Buriganga and Dhaleshwari rivers contribute significantly to atmospheric MP pollution, where emissions from vehicular traffic, industrial activities, and waste mismanagement may also release MPs into the air. Once deposited, these particles are easily mobilized by rainfall and runoff, increasing the pollutant load entering river systems. A global simulation estimated that approximately 30% of MPs generated by vehicular activity could eventually reach oceans via atmospheric pathways [141]. Given the hydrological dynamics of the Buriganga and Dhaleshwari, atmospheric MPs, once settled, are transported further through surface runoff, increasing the microplastic load in these already polluted rivers.

##### River Transport

The Buriganga and Dhaleshwari rivers are major conduits for the movement of MPs from land-based activities to downstream environments, ultimately feeding into the Bay of Bengal. These rivers drain Dhaka’s highly urbanized and industrialized areas, making them vulnerable to contamination from factory effluents, household waste, stormwater runoff, and agricultural practices. As significant carriers of land-derived pollutants, rivers globally transport approximately 0.5 million tons of plastics annually, which degrade into MPs during their journey [143,144]. In Bangladesh, inadequate infrastructure for waste handling, combined with heavy industrial output, amplifies MP loading into riverine systems. The Buriganga, which is surrounded by densely packed neighborhoods and informal industrial hubs, consistently receives untreated domestic and commercial discharges. Meanwhile, the Dhaleshwari, heavily influenced by effluents from textile dyeing and tannery operations, is another significant MP contributor. Environmental variables such as current velocity, turbulence, and seasonal hydrology influence how MPs are suspended, deposited, or resuspended within the river system [59]. These processes not only affect local water quality and aquatic health but also contribute to transboundary plastic pollution, reinforcing the role of rivers as vectors in the global spread of MPs. The escalating pressure on these freshwater systems highlights the urgent requirement for integrated river basin management, emphasizing pollution reduction at the source and improved regulatory oversight.

### 4.2. Effect of Different Emerging Contaminants on the Environment

**Figure 7:**
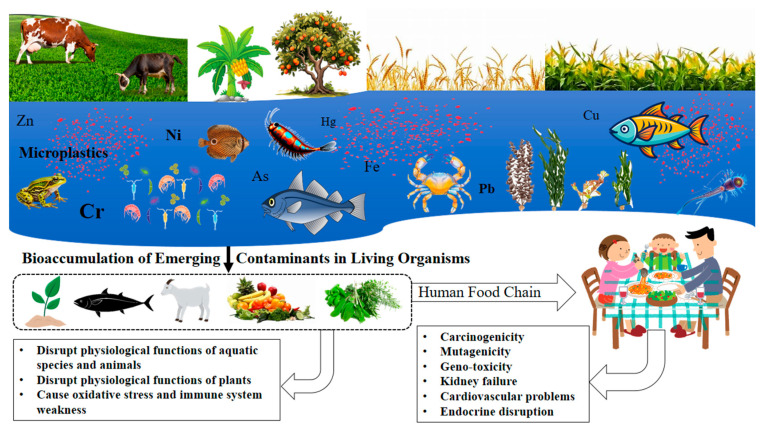
Effects of ECs on the living environment.

The effects of ECs on the Buriganga and Dhaleshwari river ecosystems, especially the living organisms like fish, plants, and humans, are shown in Figure 7. The figure delineates the transfer of HMs, MPs, and other contaminants to flora and fauna, which negatively affect the biota necessary for their everyday lives.

#### 4.2.1. Toxic Effect of Heavy Metals

While certain metals are essential to biological functions in trace amounts, their excessive accumulation can lead to severe ecological and health consequences. Aquatic organisms, including fish, often cope with metal exposure by sequestering them in inert structures (e.g., bones, scales, exoskeletons) or storing them in organs like the liver and kidneys without immediate harm [48]. However, long-term accumulation in tissues poses significant physiological threats. In Bangladesh’s Buriganga and Dhaleshwari rivers, fish species such as Chapila (*Gudusia chapra*), Batashi (*Pseudambassis baculis*), Tatkeni (*Puntius ticto*), Rohu (*Labeo rohita)*, Catla (*Catla catla)*, and Ticto barb (*Puntius ticto)* have shown elevated levels of HMs, leading to disrupted reproduction, impaired growth, and compromised organ function [24,41,46,48]. Moreover, accumulated HMs in a fish’s liver, heart, gills, and bones can cause organ damage and alter their smell, taste, and behavior [26]. For instance, fish exposed to chromium exhibit changed behaviors, including irregular swimming, cessation of food consumption, and rapid opercular movement [145]. They may also have immune system weaknesses, gill epithelial hypertrophy, and paraplegia [145]. Furthermore, zebrafish (Danio rerio) studies have revealed abnormal movements, mucus secretion, gasping openings, and color alterations [146]. These bioaccumulated metals enter the food web, increasing exposure risks for human consumers.

In urban areas like Dhaka, where fish are a dietary staple, consuming contaminated aquatic species represents a critical public health issue. Elevated Cr, Cd, and Pb levels in fish from the Buriganga indicate significant human exposure risks [34,41]. Ingesting such metals through fish or contaminated water facilitates their accumulation in vital organs. Most importantly, when the reference dose (RfD) of daily exposure to HMs is exceeded, there is a high risk of toxic effects from the corresponding metals. The RfD values displayed in Table 6 delineate the maximum permissible limit for daily human intake. High Cr (VI) exposure has been linked to carcinogenesis, reproductive impairment, and damage to the respiratory, renal, and hepatic systems [18,73,147]. The carcinogenic potential of Cd has also been confirmed; Cd interferes with DNA synthesis and protein formation, leading to systemic toxicity and cancer (Table 6) [45,148]. Furthermore, Cd exposure is particularly harmful to women, as it has a high rate of accumulation in the blood, urine, and kidneys, inducing organ dysfunction, metabolic disruption, and hormonal interaction, with additional risks of transfer to the fetus and to infants through breast milk [148,149]. Other metals also exhibit serious toxicological profiles. Hg exposure can induce altered concentration, vision issues, and unstable motion, especially in fetuses [74,149]. Chronic bronchitis, respiratory cancer, lung cancer, asthma, and sinus issues can all be caused by Ni exposure [70]. Moreover, Ni damages the kidneys, resulting in chromosome damage, mutagenesis, pulmonary cell hyperplasia, bronchial inflammation, severe renal injury, and congestion (Table 6) [70,107]. In addition, Pb, both at high and low concentrations, can cause headaches, muscle tremors, shallowness, irritability, reduced attention span, memory decline, hallucinations, and reproductive system damage [37,69,107,149]. On top of that, prolonged exposure to As can negatively impact nearly all organ systems, including cardiovascular, skin, nervous, hepatic, kidney, respiratory, and genitourinary systems [10,107,149].

Plants are affected by HMs, especially vegetables grown in contaminated soil or irrigated with polluted water. Factors influencing metal uptake include soil chemistry, water quality, plant species, and metal-binding capacity. Soil chemistry describes the chemical substances that directly impact environmental quality, plant development, and fertility. Several factors, including pH, organic matter, total nitrogen, phosphorus, and certain metal ions, are linked to it. Studies have shown that riverside plants such as *Cynodon dactylon* and *Eichhornia crassipes* near the Dhaleshwari accumulate excessive Cr, Cd, and Pb, as indicated by their high bioaccumulation (BAF), bioconcentration (BCF), and translocation (TF) factors [29].

The bioconcentration factor (BCF) of various heavy metals in Buriganaga and Dhaleshwari plant species exhibited notable variations across different metals, as presented in Table 7. For instance, Enhydra fluctuens from the Lemnoideae family demonstrated a BCF values of 0.0241 and 0.0998 for Cr and Ni, respectively. In contrast, no significant uptake was observed for Hg, As, and Mn [36,85]. Similarly, the species *Amaranthus cruentus* and *Spinacia oleracea* exhibited variable BCF values across different metals, with the BCF values for Pb and Zn ranging from 0.01 to 0.718 and 0.01 to 0.678, respectively [37]. These differences in BCF values indicate the varying abilities of plants in different species to accumulate specific metals, which could have implications for the phytoremediation potential in contaminated aquatic environments. However, the BCF values in the studies showed a lower bioconcentration of less than 1. Such a BCF could pose a risk to herbivores, consumers, and humans, leading to minimal toxic exposure. On the other hand, species with low BCF values have a lower capacity for metal uptake, possibly serving as less competent candidates for phytoremediation efforts.

Plants with high levels of Cr have decreased stem extension, absorption of water and nutrients, and plant height due to decreased nutrient flow [150]. For example, *Panicum miliaceum*, *Lactuca sativae*, and *Curcumas sativus* had decreased characteristics due to high Cr levels [150]. Moreover, the physiological functions of plants, especially photosynthesis, can be interfered with by Cr, impacting electron transport, CO_2_ fixation, enzyme activity, and photophosphorylation [150]. Plant metabolism is particularly disturbed by Hg, which also damages the formation of chlorophyll and anthocyanin, increasing the synthesis of ascorbic acid and causing physiological modifications in plants. Exposure to Cd decreases antioxidant levels and increases oxygen-free radical formation, leading to phytotoxic symptoms like leaf redness, brownish discoloration, root browning, and chlorosis in plant roots due to their higher Cd concentration [69]. While Ni is not a major element required for plant growth, increased Ni concentration can affect chlorophyll metabolism and induce oxidative stress, resulting in inappropriate flowering in plants via interference with the mitotic root tips [151]. On the other hand, Pb contamination results in chlorosis, obstruction of photosynthesis, inadequate development, destruction of the root system, diminished root growth, a reduced number of flowers, difficulty with mineral nutrition and water balance, and altered enzyme activity [152]. Another study on soybeans noticed that Pb poisoning results in morphological changes in leaves, such as a narrower blade, less xylem and phloem in vascular bundles, and a smaller width of those vessels [152].

**Table 6:** Toxic effects of heavy metals and their corresponding reference dose (RfD).

Heavy Metals	Reference Dosage (RfD) (mg/kg/day)	Main Toxic Effects	References
Cr (vi)	0.003	Carcinogenesis, reproductive impairment, damage to the respiratory, renal, and hepatic systems.	[16,70,141,153,154]
Cd	0.0005	DNA alteration, kidney dysfunction, metabolic disruption, and hormonal interaction.	[142,143,153,154]
Pb	0.0035	Headaches, muscle tremors, shallowness, irritability, reduced attention span, memory decline, hallucinations, and reproductive system damage.	[33,66,101,153,154]
Ni	0.02	Chronic bronchitis, respiratory cancer, lung cancer, asthma, mutagenesis, and pulmonary cell hyperplasia.	[67,101,153,154]
Zn	0.3	Ionic zinc (Zn^2+^) can be highly toxic to neurons, glial cells, and other cell types when it exceeds safe levels.	[68,153,154]
Hg as Methylmercury (MeHg)	0.0001	Vision impairments and unstable motion, especially in fetuses.	[71,143,153,154]
As	0.0003	Cardiovascular, skin, nervous, hepatic, kidney, respiratory, and genitourinary systems.	[101,143,153,154]
Mn	0.14	Amounts exceeding the RfD may cause symptoms of neurotoxicity.	[75,153,154]
Cu	0.04	Atherosclerosis, cardiovascular disease, diabetes, cancer progression, and neurological disorders.	[153,154,155]
Fe	0.7	Excessive Fe may cause homeostasis, DNA damage, myocardial infarction, coronary disease, and tissue damage.	[153,154,155]

**Table 7:** Bioconcentration factor (BCF) values different heavy metals in plant samples. The concentration ranges reflect the lowest and highest values reported in the references.

Plant Samples	BCFCr	BCFCd	BCFPb	BCFNi	BCFZn	BCFHg	BCFAs	BCFMn	BCFCu	BCFFe	References
*Enhydra fluctuens, Lemnoideae*	0.024–1	-	0.0301	-	0.0998	-	-	-	-	-	[85]
*Amaranthus cruentus*, *Spanacia oleracea*, *Corchorus capsularies*, *Lagenaria siceraria*, *Brassica juncea*, and *Impomoea aquatica*	0.04–0.07	0.01–0.09	0.01–0.03	0.00–0.02	0.01–0.10	-	-	-	0.03–0.15	-	[37]
*Amaranthus lividus*, *Lagenaria siceraria*, *Basella alba*, *Corchorus olitorius*, *Cucurbita moschata*, *Ipomoea aquatica*, *Brassica oleracea*, *Amaranthus gangeticus*, *Capsicum species*, *Spinacia oleracea*, *Trichosanthes anguina*, *Abelmoschus esculentus*, and *Solanum melongena*	0–0.0005	0–0.172	0–0.718	0–0.015	0.243–0.678	-	-	-	0.104–0.286	-	[54]

#### 4.2.2. Toxic Effects of Microplastics

MP contamination in river systems such as the Buriganga and Dhaleshwari rivers has become a critical environmental and public health issue. Studies have traced different MPs, including PS, PVC, PET, HDPE, Acrylonitrile butadiene styrene (ABS), PA, etc., in the sediment, water, and aquatic organisms of the Buriganga and Dhaleshwari rivers [43,87,156]. Various aquatic species, such as crabs, fish, and snails, can absorb these MPs, allowing them to eventually enter the human food chain. Similar trends have been observed globally, such as in China’s Yangtze River, where MP contamination has been associated with biodiversity loss and ecological imbalance [157]. The Toujiang River in China has been reported to have concentrations ranging from 911 to 3395 microplastics per cubic meter, which was attributed to the proximity of densely populated areas and sewage treatment plants [158]. Studies across European rivers like the Rhine and Danube show widespread MP contamination, especially with PE, near urban areas, highlighting rivers as major yet understudied conduits of plastic pollution from the land to the sea [159,160]. In North America’s Great Lakes region, urban stormwater runoff, particularly during intense rainfall, serves as a major MP source, highlighting how urban infrastructure influences the severity of pollution [161].

Multiple studies demonstrate how MPs affect aquatic organisms. Through trophic transfer, fish can ingest 3 to 11 times more microplastics from mysids than directly from the surrounding water, as these particles accumulate in prey and are consumed during normal feeding behavior [162]. For example, edible *Mytilus edulis* and *Mytilus galloprovincialis* have been found to contain 3–5 MP fibers per 10 g [27]. Carnivorous fish tend to accumulate fewer MPs than omnivorous species due to differences in feeding behavior and excretion capacity [163]. Zebrafish exposed to MPs had increased phenanthrene levels, potentially causing oxidative stress and immune suppression [164]. Moreover, the contamination of fish with a variety of chemicals (acenaphthylene, anthracene, benzoanthracene, benzofluorene, 2-ring PAHS, phenanthrene, benzoperylene, and 5,6-ring PAHS) have been shown to correlate with the accumulation of MPs, which affect the fish via some parameters of water quality, such as conductivity, TSS, and pH [165]. Similarly, amphibians such as frogs exhibit histopathological liver changes when exposed to MPs during their early life stages, affecting their developmental and ecological stability [166]. MP ingestion has also been documented in marine birds, with average concentrations of 11.9 particles per bird, potentially disrupting avian food webs [167].

Human exposure to MPs from the Buriganga and Dhaleshwari rivers occurs through direct and indirect pathways, primarily through fishing, drinking, food consumption, aquaculture, recreation, and agricultural irrigation [25,157]. MPs can act as vectors for toxic pollutants such as phthalates, polybrominated diphenyl ether (PBDE), nonylphenol, and bisphenol A, which are linked to cancer and various reproductive disorders in humans and other organisms [87]. WHO has flagged MPs as an emerging concern for public health due to their ubiquitous presence and unknown long-term effects [60]. MPs may also enter the human body through wind-borne particles, air depositions, farmland, wastewater treatment, synthetic garments, industrial exhausts, roadside dust, and marine particles, leading to cytotoxic effects, immunological illnesses, and breathing difficulties [25,116,119,164,168,169]. Depending on the metabolic process and sensitivity of the person, inhaled particles might result in symptoms similar to asthma, such as diffuse capillary fibrosis, chronic bronchitis, tumors with fiber inclusions, and lesions of the interalveolar septa [25,27].

### 4.3. Future Directions and Recommendations

Despite growing awareness, comprehensive data on the concentrations and distribution of ECs remain limited, particularly concerning their bioaccumulation and ecological impacts. Below are key focus areas for future research that can strengthen efforts to mitigate EC pollution in Bangladeshi urban rivers.

1. Conduct systematic assessments of seasonal fluctuations in EC levels, with emphasis on the pollutant loads from industrial effluents and urban runoff [170].
2. Evaluate how water, sediments, plants, and aquatic organisms (especially fish) are crucial to understanding the bioaccumulation and trophic transfer of ECs in river ecosystems [33].
3. Address the scarcity of data on persistent contaminants, such as PFAS, PPCPs, and EDCs, and investigate their concentrations, sources, and behavior across different environmental compartments by comparing studies on other regional rivers, which can inform broader mitigation strategies.
4. Develop sensitive detection methods and cost-effective wastewater treatment technologies tailored to the leather, textile, and chemical industries.
5. Employ simulation models and field experiments to trace PFAS sources, migration pathways, and deposition dynamics, particularly in industrial zones [57,171].
6. Investigate EC uptake in aquatic plants and fish, their movement across trophic levels, and potential risks to consumers, including humans.
7. Evaluate the long-term impacts of EC exposure on aquatic life and communities dependent on river water for drinking and subsistence. Include a special focus on MP contamination in aquatic systems, including underwater communities [156].
8. Determine MP concentrations in fish muscle tissue to assess the human health implications of contaminated seafood [156].
9. Examine how ecological and geographic factors influence MP ingestion and HM accumulation in freshwater organisms [172].
10. Assess MP absorption, retention, and physiological effects on aquatic and riparian vegetation in river ecosystems.
11. Establish a national database under the Department of Environment, Forests, and Climate Change to track sources, transport, and concentrations of ECs, MPs, and organic pollutants, particularly those linked to industrial activities.
12. Inform regulatory development and promote sustainable industrial practices to reduce EC contamination in the Buriganga and Dhaleshwari rivers [49].

## 5. Conclusions

This review highlights that the most vital rivers in Bangladesh, the Buriganga and the Dhaleshwari, are contaminated by ECs, especially HMs and MPs. These contaminants, originating mainly from untreated industrial effluents, have degraded water quality and disrupted the delicate balance of aquatic ecosystems, threatening the livelihoods of communities dependent on these rivers. The data regarding HMs (Pb, Cr, Cd, Ni, Hg, As, Zn, Mn, Fe, and Cu) in the water, sediment, plants, and fish of the Buriganga and Dhaleshwari rivers for this study were obtained through a literature review of the period 2005–2024. Based on the results, the concentrations of these metals are far above acceptable limits. Among the HMs, Cr, Cd, and Pb are found in water, sediment, and plants at alarming levels and stand out due to their toxic effects even at low concentrations. Moreover, the HM concentrations observed in fish from the Buriganga and Dhaleshwari rivers pose significant health risks to consumers. However, relatively few studies have focused on MP concentrations in these rivers. The hazards of bioaccumulation and biomagnification of the HMs pose a considerable risk to human health and welfare. Furthermore, weak regulatory enforcement, untreated sewage and municipal waste, inadequate wastewater treatment infrastructure, outdated water supply systems, and poor awareness about water use, reclamation, and safety are the main reasons for the HM and MP pollution in both of these Bangladeshi rivers. Hence, crucial steps must be taken to minimize the industrial effluent loads discharged into the Buriganga and Dhaleshwari rivers. This study can assist policymakers in developing national policies and effective mitigation strategies to address the harmful health effects of emerging contaminants in Bangladesh.

## Data Availability

No new data were created or analyzed in this study. Data sharing does not apply to this article.

## Abbreviations

PE	Polyethylene
PP	Polypropylene
PET	Polyethylene Terephthalate
ECs	Emerging Contaminants
HMs	Heavy Metals
MPs	Microplastics
PPCPs	Pharmaceuticals and Personal Care Products
PFAS	Per- and Poly-Fluoroalkyl Substances
EDCs	Endocrine-Disrupting Chemicals
ETP	Effluent Treatment Plant
CETP	Common Effluent Treatment Plant
PAHs	Polycyclic Aromatic Hydrocarbons
POPs	Persistent Organic Pollutants
BDL	Below Detection Limit
ND	Not Detected
WHO	World Health Organization
ASV	Average Share Value
TRV	Toxicity Reference Value
LEL	Lowest Effect Level
SEL	Severe Effect Level
FAO	Food And Agriculture Organization
MeHg	Methyl Mercury
ECR	Environmental Conservation Rules
PBDEs	Polybrominated Diphenyl Ethers
BPA	Bisphenol A
PVC	Polyvinylchloride
ABS	Acrylonitrile Butadiene Styrene
HDPE	High-Density Polyethylene
TDS	Total Dissolved Solids
IEDS	Industrial Effluent Discharge Standards
CE	Conformité Européenne
EU	European Union
SS	Suspended Solids
BOD	Biochemical Oxygen Demand
COD	Chemical Oxygen Demand
DO	Dissolved Oxygen
PCBs	Printed Circuit Boards
LDPE	Low-Density Polyethylene
IAs	Inorganic Arsenic
SR	Social Relevance
